# Profile and use of licit psychotropic substances in the former Rabat-Sale-Zemmour-Zaër Region (Morocco): the case of tiflet city

**DOI:** 10.11604/pamj.2018.31.133.13425

**Published:** 2018-10-23

**Authors:** Khadija Karjouh, Fatima-Zahra Azzaoui, Ahmed Ahami, Samira Boulbaroud

**Affiliations:** 1 Cognitive and Behavioral Neuroscience and Applied Nutrition Team, Faculty of Science, Ibn Tofail University, Kenitra, Morocco; 2 Polydisciplinary Faculty, Sultan My Sliman University, Beni Mellal, Morocco

**Keywords:** Psychotropic drugs, prescriptions, classification, analysis, quantitative, qualitative, Morocco

## Abstract

**Introduction:**

Psychopharmacology today faces serious challenges, especially related inappropriate drug choices, abuse and resulting side effects. The aim of this pharmacy based study in the City of Tiflet was to profile the prescription and usage patterns of psychotropic drugs.

**Methods:**

This study was conducted in the pharmacies of the City of Tiflet (Morocco). Descriptive statistics of 5125 prescriptions collected from 21 pharmacies were analysed, and summarized as means, percentages and proportions where appropriate.

**Results:**

The patterns of psychotropic drugs use were similar, compared to Western Countries, especially for anxiolytics/hypnotics, neuroleptics and anti-depressants. A poor monitoring of the treatment, and the lack of control of its side effects were major concerns. General practitioners ranked first among doctors who prescribed psychotropic drugs (48.5%), followed by psychiatrists (41.7%), and the rest of the prescriptions come from other specialists (neurologists, cardiologists, gynaecologists…). Among psychotropic drugs, anxiolytics dominated prescriptions (52.0%), followed by neuroleptics (29.0%) and anti-depressants (19.0%). Men consumed more psychotropic drugs than women (51.8% against 48.2% respectively).

**Conclusion:**

Anxiolytics/hypnotics constitute the main class of psychotropic drugs prescribed in the Tiflet City, followed by neuroleptics and anti-depressants. Nearly half of the population currently receive prescribed psychotropic drugs from general practitioners. Psychiatrists are less involved in the prescription and monitoring of these patients. This could predispose the population to addiction, drug misuse, intoxication, and at times, misdiagnosis of serious psychiatric illnesses. Our study highlights the urgency of reinforcing psychotropic prescription regulation and monitoring in Tiflet city.

## Introduction

Attempts to treat mental illness date back to as early as the 5000 BCs. Beliefs during this period insinuated that mental illness was the result of supernatural phenomena such as spiritual or demonic possession, sorcery, the evil eye or an angry deity, and so responded with equally mystical, and in some occasions brutal treatments [1]. The modern psychopharmacological treatments of mental diseases owe major credit to Jean Delay, and Pierre DENIKER, in 1952, with the discovery of Chlorpromazine and its benefits. As of today, numerous active substances are now available and classified due to their mechanisms of action [2]. The main five classes of psychotropic drugs are: Neuroleptics: which are general antipsychotic drugs for the treatment of schizophrenia, Anti-depressants: that target Mood Disorders (i.e. depression), anxiolytics: which are drugs for anxiety and emotional disorders, hypnotics: to treat sleep disorders, and thermo regulators: which are drugs prescribed in the context of maniac-depressive Psychosis and Bipolar Disorders [3]. The use of psychotropic drugs has increased throughout the world in recent times. The sharp global increase in the prevalence of stress explains most part of this growth, and is now considered to be a public health problem in European and North American Countries. Indeed, more than 11% of the French adult population has reported having regularly consumed at least one psychotropic drug for at least six months and this proportion of regular consumers is currently growing sharply with age [4]. Another study found that the annual prevalence of any psychotropic medication in youth was 6.7% in the United States (US), 2.9% in the Netherlands and 2.0% in Germany, and that the anti-depressant and stimulant prevalence were 3 or more times greater in the US than in the Netherlands and Germany, while antipsychotic prevalence was 1.5-2.2 times greater [5]. Data on the prevalence and licit psychotropic drug use patterns in Morocco are sparse. After our bibliographic searches, we identified only one study that was conducted on mental health in the Kingdom, by the Ministry of Health, among 5498 people. This study highlighted the fact that 50% of the participants had experienced at least one minor mental disorder in their lives. Mood disorders are more frequent among persons aged 15 years or more (26.5%). Major depressive episodes are more common in women than men (34.3%; 20.4% respectively). There is a prevalence of 11.3% in women for current generalized anxiety, compared to 7.7% in men [6]. The aim of this pharmacy based study in the City of Tiflet was to profile the prescription and usage patterns of psychotropic drugs. We specifically sought to describe the frequencies of use of different classes of these medicines, using a questionnaire filled by pharmacists of the City.

## Methods

A cross-sectional study was undertaken at 21 pharmacies in the City of Tiflet (former Rabat-Sale-Zemmour-Zaër Region, Morocco). Participants in pharmacies were required to complete a specific questionnaire regarding gender, age, socioeconomic level, medical history, psychotropic drugs, doctor speciality, number of drugs and type prescribed). Overall, 5125 prescriptions were collected. The work related to the manuscript was approved by the pharmacists responsible for all the pharmacies involved, including the pharmacy staff. Patients were informed of the survey and its objectives. The oral consents of patients were obtained prior the study, and anonymity and confidentiality of the data were ensured. The classification of drugs uses the International Non-proprietary Names (INN).

**Inclusion criteria:** Prescription containing at least one psychotropic drug.

**Exclusion criteria:** Prescription containing no psychotropic.

**Statistical analysis:** We processed the data on the Excel software package (Microsoft Corporation, version 2007). Mainly descriptive statistics are presented in the final report. The main statistics were summarized as means, proportions, and standard deviations. Data were finally presented as bar charts, histograms or pie charts where appropriate.

## Results

**Descriptive analysis of prescriptions:** Among all different prescriptions collected, 9.45% involved psychotropic medications.

**Figure 1:**
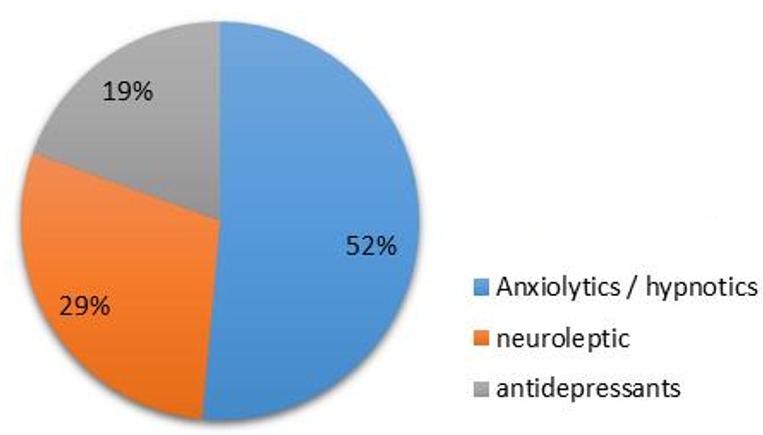
Pharmacological classification of prescribed drugs

**Pharmacological distribution of collected psychotropic drugs:** Anxiolytics/hypnotics drugs were the most prescribed drugs (52%), while neuroleptics were found in 29% prescriptions, and just 19% of these prescriptions were anti-depressants (Figure 1).

### Prescription of psychotropic drugs according to the pharmacological families

**Figure 2:**
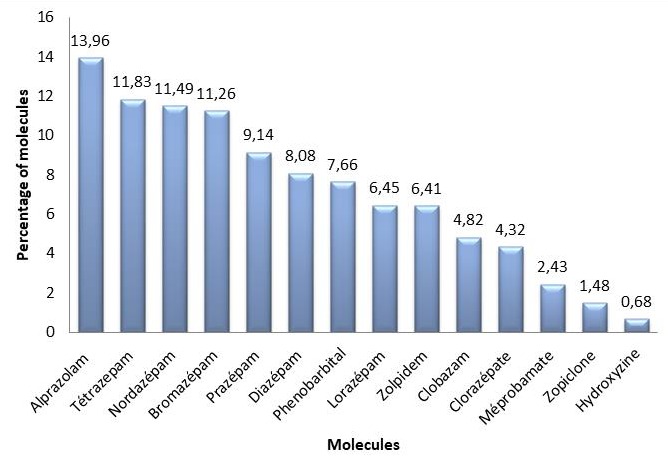
Distribution of prescribed anxiolytics and hypnotics

**The anxiolytics/hypnotics:** Alprazolam was the most commonly prescribed drug (13.96%), followed by Tetrazepam (11.83). The least prescribed of the anxiolytics was Hydroxyzine (0.68%) (Figure 2).

**Figure 3:**
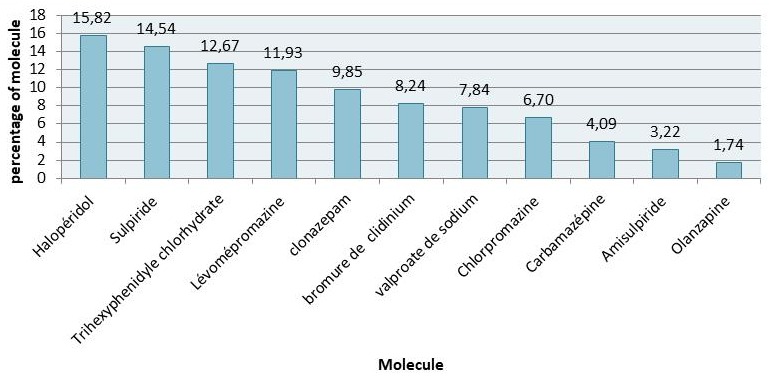
Distribution of neuroleptic prescriptions

**Neuroleptics:** The most commonly prescribed neuroleptic was Haloperidol (15.825), followed by sulpiride (14.54%). Olanzipine was the least prescribed (1.74%) (Figure 3).

**Figure 4:**
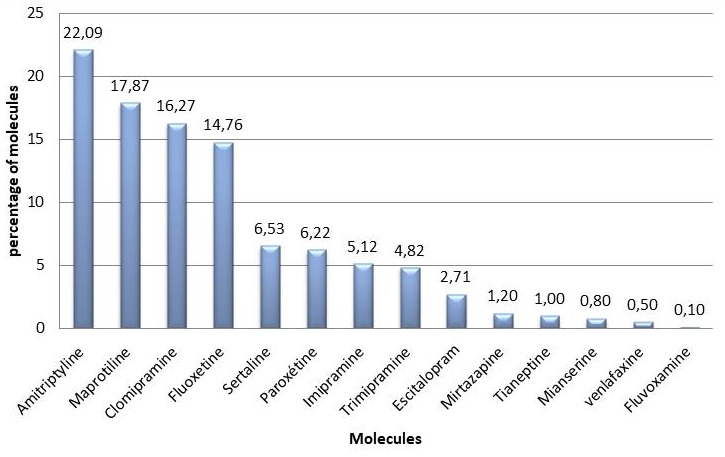
Distribution of prescribed anti-depressant drugs

**Anti-depressants:** Amitryptiline was the most prescribed anti-depressant (22.09%), followed by maprotiline (17.87%). Fluvoxamine was the least prescribed (0.10%) Figure 4.

**Prescribers:** Examining different classes of prescribing drugs made by prescribing doctors (Table 1), we found that the general practitioner prescribed 59.80% of anxiolytics/hypnotics, and 78.51% of anti-depressant drugs. In 69.30% of the cases, neuroleptics drugs were mainly prescribed by psychiatrists. However, we noticed a small percentage of prescriptions provided by other specialists (Neurologists, cardiologists, gynaecologists).

**Table 1:** Distribution of drugs types and respective prescribing doctors

Prescribing doctors	Anxiolytics/hypnotics		Neuroleptics		Anti-depressants	
	N	(%)	N	(%)	N	(%)
General practioner	1,577	59.8 %	128	8.58%	782	78.51%
Neurologists	56	2.12%	163	10.92%	12	1.2%
Psychiatrics	923	35%	1,034	69.3%	178	17.87%
Gastroenterologists	49	1.86%	74	4.96%	10	1%
Cardiologist	15	0.57%	53	3.55%	2	0.2%
Pediatricians	7	0.27%	31	2.08%	3	0.3%
Gynecologists	6	0.23%	1	0.07%	6	0.6%
Surgeons	4	0.15%	8	0.54%	3	0.3%

**Figure 5:**
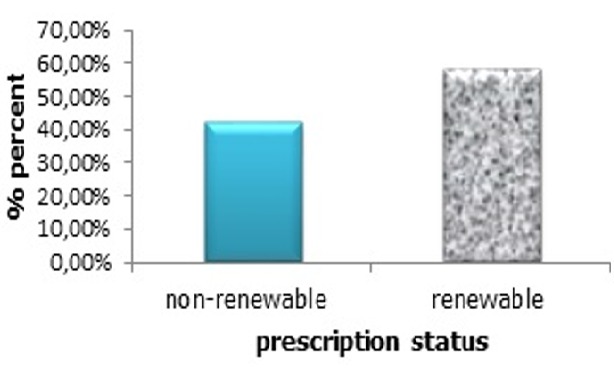
The prescription status

**Prescription status:** 57.87 % of patients had new prescriptions, and 42.13% of them used the same prescription several times, without any prior seeking of medical attention (Figure 5).

**The socioeconomic profiles of patients:** From the 5021 patients interviewed during the study, the majority were male (51.79 %), and 94.85 % were adolescent (>15 year old). Regarding the family income, 36.96% reported a minimum wage while 48.55% of patients were among low or middle socioeconomic status (Table 2).

**Table 2:** Characterization of the socio- demographic and economic profile of consumers

Socio-economic profile	N	(%)
**Gender**		
Male	2,600	51.79
Female	2,421	48.21
**Age (in years)**		
child ( ≤ 15)	259	5.15
Adult (> 15)	4,763	94,85
**Family income (wage)**		
Low	1,856	36.96
Average	2,438	48.55
High	632	12.59
Not defined	95	1.91

## Discussion

The Psychoactive medications, such as anxiolytics and anti-depressants, are the most widely prescribed drug categories in Morocco, yet few studies to our knowledge have comprehensively examined the extent, and the conditions under which psychoactive medications are prescribed. In this study, due to paucity of data in this field, we sought to characterize the usage of licit psychotropic substances in the urban area of Tifelt City situated in Rabat Sale-Zemmour-Zaer Region. The percentage of psychotropic drugs prescribed by physicians differs slightly between men and women, and these substances are widely used by adults. In contrast, Ohayon *et al* showed opposite results to ours, demonstrating that women are more exposed than men to psychotropic drugs [7]. This incoherence may be due to the fact that we did not decide to explore the use of specific therapeutic categories, and how other factors explaining psychotropic use vary by gender. Our findings showed that anxiolytic/hypnotic drugs represented (52.0%) of prescriptions, followed by narcoleptics (29.0%) and anti-depressants (19.0%). This data showed that the studied population suffers from anxiety and the anxiolytic drugs are a widely prescribed type of medication to treat anxiety symptoms in Tifelt City. Several consistent data found that anxiety disorders are the most prevalent class of mental disorders in the general population, with estimated lifetime prevalence of any anxiety disorder averaging approximately 16% across the (WMH) Surveys. There is a wide variation around these averages though, with prevalence estimates generally higher in Western Developed Countries than in Developing Countries [8-10].

According to medication class and physician specialty, our data showed that among the three studied classes, the anxiolytics and anti-depressants were prescribed mostly by general practitioners compared to other specialist physicians. To find an explanation for this finding, we must find the answer to the following questions: firstly, how requesting specific medication can meet the patient's needs, secondly, how these needs are affecting their prescription, and thirdly, if the sociodemographic status of patient provide an appropriate component to make a relevant counseling among medication types and physician specialty or not. Indeed, there is a real problem of psychotropic use in Morocco. A very large number of people consume psychotropic drugs chronically for potentially non-beneficial therapeutic purposes, as we found that 42.13% of patients used the same prescriptions more than once (normally, a prescription is valid for 6 months from the date of the prescription, unless the medicine prescribed contains a controlled drug), showing that consumers make self-diagnosis. Requests and prescriptions from their physicians are not necessary to provide them the required health care, update the appropriate diagnosis, and provide the appropriate drugs. This renders follow up of these patients by their treating doctors extremely difficult [11]. Lack of universal health coverage is a serious problem that hinders optimal medical care in Morocco. This pushes patients into auto medication and auto renewal of old prescriptions without any medical advice. Of note, most of these patients are low to medium income earners. This makes access to psychiatrists difficult, and the medical practitioners are then bound to make most of the prescriptions. One could also question the appropriateness and effective referral of patients that actually need psychiatric care to the specialist by the general practitioners. The number of psychiatrists to serve the population is generally inadequate. In a study by Lagnaoui *et al*, 5.5% of the participants among people with any mood or anxiety disorder, consumed anxiolytic drugs, especially benzodiapezines [12]. This previous report and our current study are in line, of the high risk related to exorbitant use of medication and its harmful effects (biological, psychological and behavioral) [13]. Regarding the pharmacological classes of drugs, we found that Alprazolam was widely used by people suffering from anxiety. Alprazolam is widely prescribed for people suffering from panic attacks, anxiety disorder and various social phobias [14, 15]. Although alprazolam is effective in medical settings, it can create an addictive "high" that can lead to drug abuse [16].

The most prescribed pharmacological anti-depressant molecule in our sample was amitriptyline. Amitriptyline is a tricyclic anti-depressant agent which also has analgesic properties. Major drawbacks of its use is that amitriptyline can produce side effects such as sedation [17], decrements in psychomotor performance, memory and attention, plus amitriptyline addiction [18, 19]. The most widely used antipsychotics found in our research were haloperidol and sulpiride. Haloperidol is extremely efficient in treating the positive and negative symptoms of psychoses and schizophrenia. Long-term use of the drug, however, results in an irreversible motor disorder involving the oral facial muscles and the limbs, which has been a source of major concern in the medical community [20]. A recent meta-analysis of several Randomized Controlled Trials (RCTs) reported that sulpiride had similar efficacy and fewer side effects as compared with the other Typical Antipsychotics (TAs), such as haloperidol, chlorpromazine, and perphenazine [21, 22]. We noted that the principal problem lies in the respect of prescriptions. Moreover, it is essential that consumers too should recognize and respect non-prescription medicines. However, the use of psychotropic drugs in Morocco remains more or less controllable by the strict respect of prescriptions, and prescriptions should be regularly checked by the pharmacists. Nevertheless, the main problem remains mainly in the monitoring of the various side effects of these substances, their incidents and accidents, as well as the monitoring of the patients' prognosis. Doctors have a responsibility in advising patients on the validity of a prescription, and to provide timely referrals to psychiatrists. It is important for the health care system to increase the number of trained medical personnel that are involved in mental health in the communities and health system, to help attenuate this growing burden, arising from inappropriate use of licit psychotropic substances.

## Conclusion

This research provides a broad view of the nature of psychoactive medication prescribing, which may serve as a guide to future research about these medications. Up to 50% of psychotropic drugs are prescribed by the general practitioner. Greater prevention efforts are needed to reduce the potential scope of misuse and drug abuse, and proper use of prescription medication by patients.

### What is known about this topic

- The consumption of psychotropic drugs is a public health problem;
- The use of psychotropic drugs has become very rampant in Morocco;
- There is an increase in the trivialization and use of psychotropic drugs.

### What this study adds

- Out of ten medications issued, one of them turns out to be psychotropic;
- Most psychotropic drugs in the city of Tiflet are prescribed by the general practitioner;
- Psychotropic drug use patterns in Morocco are in concordance with reports in the literature.

## Competing interests

The authors declare no competing interests.
